# The longitudinal relationship between physical capacity and self-perceived physical functioning after total hip and knee arthroplasty

**DOI:** 10.1016/j.ocarto.2026.100880

**Published:** 2026-09-23

**Authors:** Dennis J. van den Berg, Esther T. Maas, Thea P.M. Vliet Vlieland, Max Reijman, Henri Kiers, Raymond W.J.G. Ostelo, Maaike G.J. Gademan, PaTIO Study group, Dominique C. Baas, Roos Bazuin, Kirsten D.S. Boerma-Argelo, Bert Boonen, Koen P.K. Bos, Tim A.E.J. Boymans, Ellen A. Breedveld, Martine de Bruijn, Baukje Dijkstra, Jordy Elings, Arthur de Gast, Taco Gosens, Dirk J. Hofstee, Rob P.A. Janssen, Paulien M. van Kampen, Herman H. Kaptijn, Sander Koëter, Keetie C.A.L.C. Kremers-van de Hei, Wai-Yan Liu, Ton A.F. Lenssen, Micheal F. Nieboer, Lotte Nieuwenhuys-Kroon, Peter A. Nolte, Julia C.A. Noorduyn, Ellen Oosting, Jantsje H. Pasma, Rudolf W. Poolman, Marjolein Schager, Martijn G.M. Schotanus, Remko J.A. Sonnega, Martin Stevens, Hennie Verburg, Suzan H.M. Verdegaal, Wierd P. Zijlstra

**Affiliations:** h Tergooi Medical Center, Department of Orthopaedic Surgery, Laan van Tergooi 2, Hilversum, 1212 VG, the Netherlands; i Haga Hospital, Department of Orthopedic Surgery, Sportlaan 600, The Hague, 2566 MJ, the Netherlands; j Northwest Clinics, Centre for Orthopaedic Research Alkmaar (CORAL), Wilhelminalaan 12, Alkmaar, 1815 JD, the Netherlands; k Zuyderland Hospital, Department of Orthopedic Surgery and Traumatology, Dr. H. van der Hoffplein 1, Sittard-Geleen, 6162 BG, the Netherlands; l Erasmus MC, University Medical Center Rotterdam, Department of Orthopedics and Sports Medicine, P.O. Box 2040, Rotterdam, 3000 CA, the Netherlands; m University MC+ Maastricht, Department of Orthopedic Surgery, P. Debyelaan 25, Maastricht, 6229 HX, the Netherlands; n Gelderse Vallei Hospital, Department of Physiotherapy, Willy Brandtlaan 10, Ede, 6716 RP, the Netherlands; o Medical Center Leeuwarden, Department of Orthopedic Surgery, Henri Dunantweg 2, Leeuwarden, 8934 AD, the Netherlands; p Diakonessenhuis, Department of Physiotherapy, Bosboomstraat 1, Utrecht, 3582 KE, the Netherlands; q Diakonessenhuis, Department of Orthopedic Surgery, Bosboomstraat 1, Utrecht, 3582 KE, the Netherlands; r Diakonessenhuis, Clinical Orthopedic Research Center-mN, Jagersingel 1, Zeist, 3707 HL, the Netherlands; s Elisabeth-Tweesteden Hospital, Department of Orthopedic Surgery, Hilvarenbeekse Weg 60, Tilburg, 5022 GC, the Netherlands; t Máxima Medical Center, Department of Orthopaedic Surgery and Trauma, Dominee Theodor Fliednerstraat 1, Eindhoven, 5631 BM, the Netherlands; u Orthopaedic Biomechanics, Department of Biomedical Engineering, Eindhoven University of Technology, the Netherlands; v Fontys University of Applied Sciences, Eindhoven, the Netherlands; w Bergman Clinics Bewegen Rijswijk, Department of Orthopaedic Surgery, Braillelaan 10, Rijswijk, 2289 CM, the Netherlands; x Lange Land Hospital, Department of Orthopaedic Surgery, Toneellaan 1, Zoetermeer, 2725 NA, the Netherlands; y Canisius Wilhelmina Hospital, Department of Orthopedic Surgery, Weg door Jonkerbos 100, Nijmegen, 6532 SZ, the Netherlands; z University MC Maastricht, Department of Physiotherapy, P. Debyelaan 25, Maastricht, 6229 HX, the Netherlands; aa Zuyd Hogeschool, Department of Physiotherapy, P. Debyelaan 25, Maastricht, 6229 HX, the Netherlands; ab Alrijne Hospital, Department of Orthopedic Surgery, Simon Smitweg 1, Leiderdorp, 2353 GA, the Netherlands; ac Spaarne Gasthuis Hospital, Department of Orthopaedic Surgery, Spaarnepoort 1, Hoofddorp, 2134 TM, the Netherlands; ad Department of Oral Cell Biology, Academic Centre for Dentistry (ACTA), the Netherlands; ae University of Amsterdam and Vrije Universiteit Amsterdam, Gustav Mahler 3004, Amsterdam, 1082 LA, the Netherlands; af OLVG Amsterdam, Department of Orthopaedic Surgery, Joint Research, Oosterpark 9, Amsterdam, 1091 AC, the Netherlands; ag Sports Valley, Department of Physiotherapy, Gelderse Vallei Hospital, Willy Brandtlaan 10, Ede, 6716 RP, the Netherlands; ah LUMC, Leiden University Medical Center, Department of Orthopedic Surgery, P.O. Box 9600, Leiden, 2300 RC, the Netherlands; ai University of Groningen, University Medical Center Groningen, Department of Orthopedic Surgery, Hanzeplein 1, Groningen, 9713 GZ, the Netherlands; aj Reinier de Graaf Hospital, Department of Orthopedic Surgery, Reinier de Graafweg 5, Delft, 2625 AD, the Netherlands; a Department of Health Sciences, Faculty of Science, Vrije Universiteit Amsterdam, Amsterdam Movement Sciences, the Netherlands; b Institute for Movement Studies, University of Applied Sciences Utrecht, Utrecht, the Netherlands; c Department of Orthopaedics, Rehabilitation and Physiotherapy, Leiden University Medical Center, Leiden, the Netherlands; d Basalt Rehabilitation, Leiden, the Netherlands; e Department of Orthopaedics & Sport Medicine, Erasmus MC, Rotterdam, the Netherlands; f Department of Epidemiology and Data Science, Amsterdam UMC, Location Vrije Universiteit, Amsterdam Movement Sciences Research Institute, the Netherlands; g Department of Clinical Epidemiology, Leiden University Medical Center, Leiden, the Netherlands

**Keywords:** Total hip arthroplasty, Total knee arthroplasty, Physical functioning, Patient reported-outcomes, Random intercept cross-lagged panel model, Postoperative rehabilitation

## Abstract

**Objective:**

To examine longitudinal within-person effects between capacity and self-perceived performance during postoperative physiotherapy in patients with total hip and knee arthroplasty (THA/TKA).

**Methods:**

We performed a secondary analysis of a cluster randomized controlled trial on physiotherapy after THA and TKA using random intercept cross lagged panel models. Capacity was assessed with the 40-m fast-paced walk test (40 m FPWT) and the 30-s chair-stand test (30-s CST), and self-perceived performance with the Oxford Hip/Knee Score functioning subscale (OHS/OKS) and the Hip disability/Knee injury and Osteoarthritis Outcome Score–Physical function Short form (HOOS-PS/KOOS-PS). Assessments were performed preoperatively and at 3 and 12 months postoperatively.

**Results:**

312 THA (mean age 66, SD: 9; 53% female) and 312 TKA (mean age 67, SD: 8; 55% female) were included. From pre-surgery to 3 months, no effects were found between capacity and self-perceived performance in either THA or TKA.

From 3 to 12 months, in THA, 40 m FPWT performance at 3 months had a positive effect (β 0.34, 95% CI 0.05–0.63**)** on HOOS-PS scores at 12 months. In TKA, 40 m FPWT performance at 3 months had a positive effect on both OKS functioning (β 0.24 95% CI 0.05–0.43**)** and KOOS-PS (β 0.29 95% CI 0.11–0.47**)** scores at 12 months. No other effects were observed.

**Conclusion:**

From pre-surgery to 3 months, capacity and self-perceived performance showed no effects within individuals. However, during the early postoperative phase, gait speed may be a relevant treatment target to improve long-term self-perceived performance.

## 1 Introduction

Total hip and total knee arthroplasty (THA/TKA) are effective treatment procedures for end-stage hip and knee osteoarthritis, ranking among the most frequently performed surgeries worldwide [1]. A considerable proportion of patients, up to 95%, receive post-discharge physiotherapy [2,3]. Several physiotherapy [[4], [5], [6], [7]], orthopaedic [8,9] and outcome measurement [10,11] guidelines recognize physical functioning as an important outcome following THA and TKA and recommend assessing it using patient reported outcome measures (PROMs) [[7], [8], [9], [10]], performance-based tests [11] or a combination of both [[4], [5], [6]].

While both PROMs and performance-based tests can be used to assess physical functioning, they capture different aspects of the construct [12]. PROMs for physical functioning reflect the self-perceived performance of activities and functions referred to as ‘performance’ in the International Classification of Functioning, Disability, and Health (ICF) [13]. Performance-based tests reflect one's ability to execute a task (e.g. rising from a chair) at maximum capacity in a standardized environment, referred to as ‘capacity’ in the ICF. The mechanism underlying improvements in these performance-based test outcomes (i.e. one's capacity), such as gains in strength and joint mobility, are relatively well understood [14,15]. However, it remains unclear whether such improvements in capacity lead to corresponding improvements in patients' self-perceived performance or vice versa. A better understanding of whether and how capacity and self-perceived performance influence each other over time could help identify meaningful targets for rehabilitation following THA and TKA.

Several studies compared capacity and self-perceived performance after THA and TKA and consistently suggest these constructs develop independently over time. For THA, longitudinal analyses revealed no correlations between changes in subscales of the Hip disability and Osteoarthritis Outcome Score (HOOS) and performance tests (Timed Up and Go, stair-climbing, 6-min walk) [16], and cross-sectional analyses at one year revealed no HOOS differences between patients classified as “improved” versus “unchanged” on the Five Times Sit to Stand, Timed Up and Go, and Single Limb Mini Squat tests [17]. For TKA, Knee injury and Osteoarthritis Outcome Score (KOOS) activities of daily living subscale scores did not correlate with changes in 6-min walk distance [18], and early declines in performance tests (6-min walk, Stair Climbing, Timed Up and Go) were not reflected in matched PROM items [19]. Similar dissociations between capacity and self-perceived performance were observed in mixed THA/TKA cohorts during the first postoperative weeks [20] and by 4 months [20,21]. However, these studies used between-person analyses, not distinguishing within-individual changes from between-individual differences. For example, a between-individual relationship may indicate that people with higher average capacity generally perceive their physical performance as better, whereas a within-individual relationship indicates that a change in an individual's capacity is accompanied by a (subsequent) change in their perceived performance. Distinguishing these effects helps clinicians determine whether focusing treatment on improving capacity is likely to translate into improved perceived performance or vice versa, thereby informing treatment goals and clinical decision-making. To our knowledge, no study has used longitudinal models that address this limitation, such as the random intercept crossed lagged panel model (RI-CPLM) [22], to assess how capacity and self-perceived performance interact within individuals over time after THA and TKA.

To build on existing knowledge and address these gaps, this study aimed to estimate the within-person bidirectional effects between performance-based test outcomes (i.e. capacity) and PROM outcomes (i.e. self-perceived performance) during postoperative physiotherapy in patients with THA and TKA. A better understanding of these relationships can help identify clinically meaningful targets for rehabilitations following THA and TKA.

## 2 Methods

### 2.1 Design

This study was a secondary analysis of a multicentre randomized controlled clinical trial: PhysiotherApeutic Treat-to-target Intervention after Orthopaedic surgery; A cost-effectiveness study (PaTIO-study) [23]. The PaTIO-study aimed to evaluate the cost-effectiveness of an optimized, treat-to-target personalized physiotherapy strategy in TKA and THA patients compared to usual postoperative physiotherapy [24]. The study protocol was approved by the Erasmus MC Medical Ethics Committee (registration code NL61763.078.17) and was registered in the Dutch Trial Registry (registration number NL-OMON55756). The detailed protocol [24] and the results [23] of the PaTIO-study are reported elsewhere.

### 2.2 Participants

The PaTIO study included patients scheduled for a primary THA or TKA. Eligibility criteria included: clinical and radiological knee or hip osteoarthritis, scheduled for a primary THA or TKA and willingness to comply with the study protocol. Exclusion criteria were: scheduled for THA or TKA not due to osteoarthritis, uncontrolled cardiovascular disease or hypertension, a history of neuromuscular disorder that affects lower extremity function, a terminal illness, a psychiatric disorder, planned contralateral joint replacement during study follow-up, inability to attend follow-up measurements or postoperative physiotherapy in primary care, or insufficient Dutch proficiency. All participants provided written informed consent.

For the present analyses, data from patients in the intervention and control groups were pooled as there were no significant differences in any of the outcomes [23], allowing for a unified analysis of within-subject change for both the THA and TKA groups.

### 2.3 Measures

PROMs were administered pre-surgery and at 6 weeks and 3, 6, 9 and 12 months post-surgery. Performance-based tests were conducted by trained assessors at the participating hospitals before surgery and at 3 and 12 months post-surgery assessments. In the present study T1 was defined as the pre-surgical assessment, T2 as the 3-months post-surgery assessment and T3 as 12-month post-surgery assessment.

#### 2.3.1 Capacity

Capacity was assessed using performance-based tests including the 40 m fast paced walk test (40 m FPWT) and the 30-s chair-stand test (30-s CST). These tests are part of the minimal core set recommended by the Osteoarthritis Research Society International for both research and clinical practice [25]. The 40 m FPWT test assesses the walking speed over short distances. Maximal walking speed is scored on marked walkway of 10 m, which is repeated four times. The result is expressed as speed m/s by dividing the total distance (40 m) by the total time it took to complete the four trials [26]. The 30-s CST test assesses the time needed to stand 10 times from a standard chair and reflects an individual's lower extremity strength [27]. Although the PaTIO-study included the Stair Climbing Test, challenges in standardising the assessment across test sites introduced measurement inconsistency, making the scores unsuitable for our analysis.

#### 2.3.2 Self-perceived performance

In line with international recommendations on the use of PROMs in patients with THA or TK [10,28], the Oxford Hip/Knee Score [29,30] (OHS/OKS) and the HOOS/KOOS Physical functioning Shortform [31,32] (HOOS-PS/KOOS-PS) were administered to assess self-perceived performance.

The OHS and OKS assess pain and function following THA and TKA. As only physical functioning was of interest, the physical functioning component score was calculated as described elsewhere [33,34]. For the present analysis, raw scores were used to preserve model stability and ensure convergence. For the OHS function subscale, scores ranged from 0 (extreme disability) to 24 (no disability), and for the OKS function subscale, scores ranged from 0 (extreme disability) to 20 (no disability).

The HOOS-PS and KOOS-PS are self-reported questionnaires which assess physical functioning in people with a hip or knee complaints including joint arthroplasty. As with the OHS/OKS, raw scores were used for the analysis, which ranged from 0 (no disability) to 20 (extreme disability) for the HOOS-PS and 0 (no disability) to 28 (extreme disability) for the KOOS-PS.

#### 2.3.3 Sociodemographic and clinical characteristics

Baseline sociodemographic and clinical characteristics included age (years), sex (female/male), body mass index (BMI), living status (alone/together), pain during activity using the Numeric Rating Scale (0 no pain, 10 extreme pain), American Society of Anaesthesiology (ASA) classification (I: a normal healthy patient, II: a patient with mild systemic disease, III: a patient with severe systemic disease), and duration of complaints (years) [35].

### 2.4 Statistical analysis

Statistical analyses were performed using R (v4.5.0, R Foundation for Statistical Computing, Vienna, Austria). Sociodemographic and clinical characteristics were summarized using frequencies and percentages for categorical variables, and means and standard deviations for continuous variables.

Where appropriate, Pearson or Spearman correlation coefficients were calculated for all capacity tests and measures of self-perceived performance at each time point to provide an initial overview of correlations between capacity and self-perceived performance. Correlation coefficients of 0.10–0.29 were considered small, 0.30–0.49 moderate, and ≥0.50 large [36]. To evaluate the overall change in outcomes, scores at T1 were subtracted from scores at T3, and summarized using means and standard deviations.

To examine how capacity and self-perceived performance influence each other over time within patients a RI-CLPM was used (Fig. 1) [37]. The RI-CLPM helps to understand these relationships by separating variance into stable differences between people from differences within the same person over time [37]. Stable differences between people, such as age, sex, and socio-economic status, may confound the relationship between capacity and self -perceived performance and are captured as random intercepts (green dotted arrows Fig. 1). For example, on average older people may walk slower than younger people. By accounting for these differences, the model separates these potential sources of confounding from changes occurring within individuals over time.

**Fig. 1:**
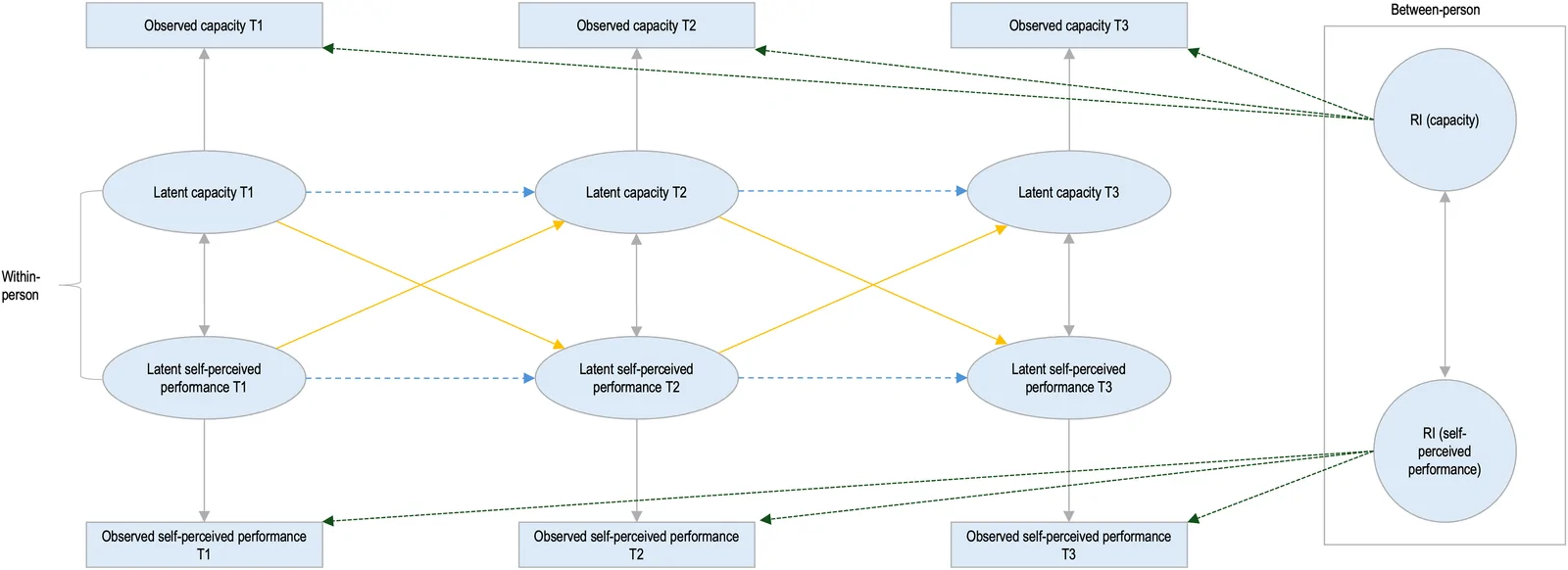
**Example path diagram of Random intercept crossed-lagged panel model** Figure legend. T1 = pre-surgery, T2 = 3 months post-surgery, T3 = 12 months post-surgery, RI = random intercept.

Differences within the same person are represented as latent variables (oval circles Fig. 1). These reflect the difference between an individual's result at a specific assessment (T1, T2 or T3) and their personal average (i.e. the personal average of T1, T2 and T3 estimated by the model for that specific individual). When an individual's score at a specific assessment is higher than their personal average, this reflects better than personal average capacity or self-perceived performance. The model then tests whether these within-person differences persist over time through autoregressive paths (blue dashed arrows Fig. 1). For example, if an individual walks faster than their personal average speed at T2, the model tests if this difference carries over to T3.

Most importantly, the RI-CLPM estimates cross-lagged effects (solid yellow lines in Fig. 1). These effects show whether a difference in one measure, such as capacity, at an earlier assessment is followed by a difference in another measure, such as self-perceived performance, at a later assessment. For example, a positive cross-lagged effect from physical capacity at T2 to self-perceived performance at T3 would indicate that an individual who performs better than their personal average capacity at 3 months post-surgery will generally subsequently report self-perceived performance above their personal average at 12 months post-surgery. Because these effects are examined within the same patient, they are not influenced by stable characteristics like age or sex and reflect potential causal relationships.

#### 2.4.1 Missing data

To be included in the sample for analysis, participants had to have a least one valid measure of capacity or self-perceived performance at any assessment. Full-information maximum likelihood estimation was used to handle missing data across all assessments, under the assumption that data were missing at random. To increase precision and reduce potential bias [38], demographic and clinical characteristics were included as predictors of the observed outcomes at each assessment, with time-invariant effects, as described by Mulder and Hamaker [39]. These included age, sex, BMI, ASA-score, pre-operative pain during activity, and living status, all of which have been associated with worse self-perceived performance following THA [[40], [41], [42], [43], [44]] and TKA [[41], [42], [43], [44]]. The missing-at-random assumption was checked by comparing baseline characteristics and outcomes between participants with complete versus incomplete data using frequencies and percentages.

## 3 Results

### 3.1 Participant characteristics

All 624 (312 THA, 312 TKA) of the original trial participants had at least one valid measure of capacity or performance and were included in the present analysis. Complete 12-month data were available for 158 (51%) THA and 143 (46%) TKA participants. The COVID-19 pandemic contributed substantially to data incompleteness, as public health restrictions temporarily prevented performance-based testing. Missing data patterns appeared at random, with exploratory analyses showing no differences between complete and incomplete cases across outcome measures, clinical, or demographic characteristics (Supplementary file 1).

On average, participants in the THA group were 66.6 (SD: 8.9) years old, with 53% being female and 22% living alone. Furthermore, participants who underwent THA had, on average, 4.9 years (SD: 5.8) of complaints prior to surgery and a mean baseline Numeric Pain Rating Scale score for pain during activity of 3.9 (SD: 3.0). Participants in the TKA group were, on average, 66.9 (SD: 8.2) years old; 55% were female, and 17% of the group lived alone. On average, participants who underwent TKA had 9.9 years (SD: 9.1) of complaints prior to surgery and a mean baseline Numeric Pain Rating Scale score of 6.7 (SD: 2.0). A full overview of participant characteristics is provided in Table 1.

**Table 1:** Demographic characteristics and outcomes at baseline of individuals undergoing a total hip/knee arthroplasty taking part in the PaTIO-study.

	Total hip arthroplasty (n = 312)	Total knee arthroplasty (n = 312)
Age, years, mean (SD)	66.6 (8.9)	66.9 (8.2)
*Missing (n)*	*2*	*3*
Sex, female, n (%)	165 (53)	169 (55)
*missing (n)*	*2*	*3*
BMI, mean (SD)	27.1 (4.5)	29.4 (4.9)
*Missing (n)*	*2*	*3*
Living status, living alone, n (%)	67 (22)	52 (17)
*missing (n)*	*8*	*7*
Years of complaints, mean (SD)	4.9 (5.8)	9.9 (9.1)
*Missing (n)*	*8*	*7*
ASA classification, n (%)		
I	60 (19)	53 (17)
II	191 (62)	189 (61)
III	55 (18)	68 (22)
*Missing (n)*	*6*	*2*
Pain during activity (0-10), mean (SD)	3.9 (3.0)	6.7 (2.0)
*Missing (n)*	*8*	*5*
HOOS-PS/KOOS-PS score, mean (SD)	10.1 (3.8)	13.2 (5.9)
*Missing (n)*	7	*5*
OHS/OKS function subscale score, mean (SD)	15.3 (8.3)	12.6 (3.8)
*Missing (n)*		
40 m FPWT, m/s, mean (SD)	1.2 (0.3)	1.1 (0.3)
*Missing (n)*	*9*	*17*
30-s CST, repeats, mean (SD)	9.9 (3.4)	9.6 (3.4)
*Missing (n)*	*8*	*14*

Table legend. n = number, BMI = body mass index, SD = standard deviation, ASA = American Society of Anaesthesiologists, HOOS-PS/KOOS-PS = Hip Disability/Knee Injury Osteoarthritis Outcome Score, OHS/OKS = Oxford Hip/Knee Score, 40 m FPWT = 40-m Fast Paced Walking Test, 30-s CST = 30-s Chair Stand Test.

### 3.2 Correlations across all outcome measures at all time points

In the THA group, gait speed and Chair rise performance demonstrated strong temporal stability, with correlations ranging from *ρ* = 0.56 to 0.70 across time points. Self-perceived performance also showed moderate correlations over time (*ρ* = 0.35 to 0.39). Correlations between capacity and self-perceived performance were small to moderate, ranging from *ρ* = 0.29 to 0.42 (Table 2).

**Table 2:** Correlation matrix of outcome measures in patients undergoing a total hip arthroplasty.

*r*	40 m FPWT			30-s CST			OHS function			HOOSPS		
**Assessment**	T1	T2	T3	T1	T2	T3	T1	T2	T3	T1	T2	T3
40 m FPWT T1	1											
40 m FPWT T2	0.70^b^	1										
40 m FPWT T3	0.60^b^	0.76^b^	1									
30-s CST T1	0.59^b^	0.42^b^	0.31^b^	1								
30-s CST T2	0.53^b^	0.61^b^	0.50^b^	0.66^b^	1							
30-s CST T3	0.50^b^	0.57^b^	0.56^b^	0.56^b^	0.72^b^	1						
OHS function T1	0.42^b^	0.35^b^	0.28^b^	0.37^b^	0.21^b^	0.28^b^	1					
OHS function T2	0.28^b^	0.38^b^	0.26^b^	0.35^b^	0.35^b^	0.39^b^	0.39^b^	1				
OHS function T3	0.34^b^	0.38^b^	0.38^b^	0.33^b^	0.31^b^	0.39^b^	0.37^b^	0.59^b^	1			
HOOSPS T1	0.29^b^	0.19^b^	0.11	0.3^b^	0.17^a^	0.15^a^	0.73^b^	0.33^b^	0.31^b^	1		
HOOSPS T2	0.28^b^	0.38^b^	0.25^b^	0.25^b^	0.34^b^	0.35^b^	0.37^b^	0.74^b^	0.48^b^	0.35^b^	1	
HOOSPS T3	0.31^b^	0.37^b^	0.3^b^	0.27^b^	0.31^b^	0.34^b^	0.3^b^	0.53^b^	0.74^b^	0.38^b^	0.56^b^	1

Table legend.

a p < 0.05.

b p < 0.01. HOOS-PS = Hip Disability Injury Osteoarthritis Outcome Score, OHS = Oxford Hip Score, 40 m FPWT = 40-m Fast Paced Walking Test, 30-s CST = 30-s Chair Stand Test.

In the TKA group, similar patterns were observed, with gait speed and lower extremity strength showing moderate to strong stability over time (*ρ* = 0.47 to 0.62). Self-perceived performance exhibited moderate correlations over time (*ρ* = 0.34 to 0.47). Correlations between capacity and self-perceived performance were small to moderate, ranging from *ρ* = 0.22 to 0.42 (Table 3).

**Table 3:** Correlation matrix of outcome measures in patients undergoing a total knee arthroplasty.

*r*	40 m FPWT			30-s CST			OHS function			KOOSPS		
**Assessment**	T1	T2	T3	T1	T2	T3	T1	T2	T3	T1	T2	T3
40 m FPWT T1	1											
40 m FPWT T2	0.63^a^	1										
40 m FPWT T3	0.62^a^	0.83^a^	1									
30-s CST T1		0.45^a^	0.3^a^	1								
30-s CST T2	0.39^a^	0.57^a^	0.51^a^	0.50^a^	1							
30-s CST T3	0.43^a^	0.58^a^	0.66^a^	0.47^a^	0.71^a^	1						
OKS function T1	0.38^a^	0.32^a^	0.15	0.42^a^	0.25^a^	0.13	1					
OKS function T2	0.20^a^	0.35^a^	0.10	0.30^a^	0.36^a^	0.15^a^	0.45^a^	1				
OKS function T3	0.25^a^	0.37^a^	0.27^a^	0.32^a^	0.38^a^	0.28^a^	0.47^a^	0.68^a^	1			
KOOSPS T1	0.27^a^	0.21^a^	0.06	0.37^a^	0.21^a^	0.06	0.77^a^	0.39^a^	0.39^a^	1		
KOOSPS T2	0.11	0.22^a^	0.07	0.23^a^	0.30^a^	0.11	0.30^a^	0.73^a^	0.51^a^	0.34^a^	1	
KOOSPS T3	0.22^a^	0.37^a^	0.28^a^	0.30^a^	0.36^a^	0.27^a^	0.40^a^	0.63^a^	0.83^a^	0.40^a^	0.59^a^	1

Table legend.

a p < 0.01.KOOS-PS = Knee Injury Osteoarthritis Outcome Score, OKS = Oxford Knee Score, 40 m FPWT = 40-m Fast Paced Walking Test, 30-s CST = 30-s Chair Stand Test.

### 3.3 Changes in capacity and self-perceived performance

Fig. 2 shows the average change and individual trajectories for the primary outcomes in the THA group. From pre-surgery to the 12-month follow-up, patients improved on average by 32% (0.24 m/s, SD 0.29) in gait speed on the 40 m FPWT, by 43% (3.4 repetitions, SD 3.9) on the 30-s chair-stand test, by 67% (6.5 points, SD 4.6) on the OHS function subscale, and by 68% (6.8 points, SD 4.0) on the HOOS-PS.

**Fig. 2:**
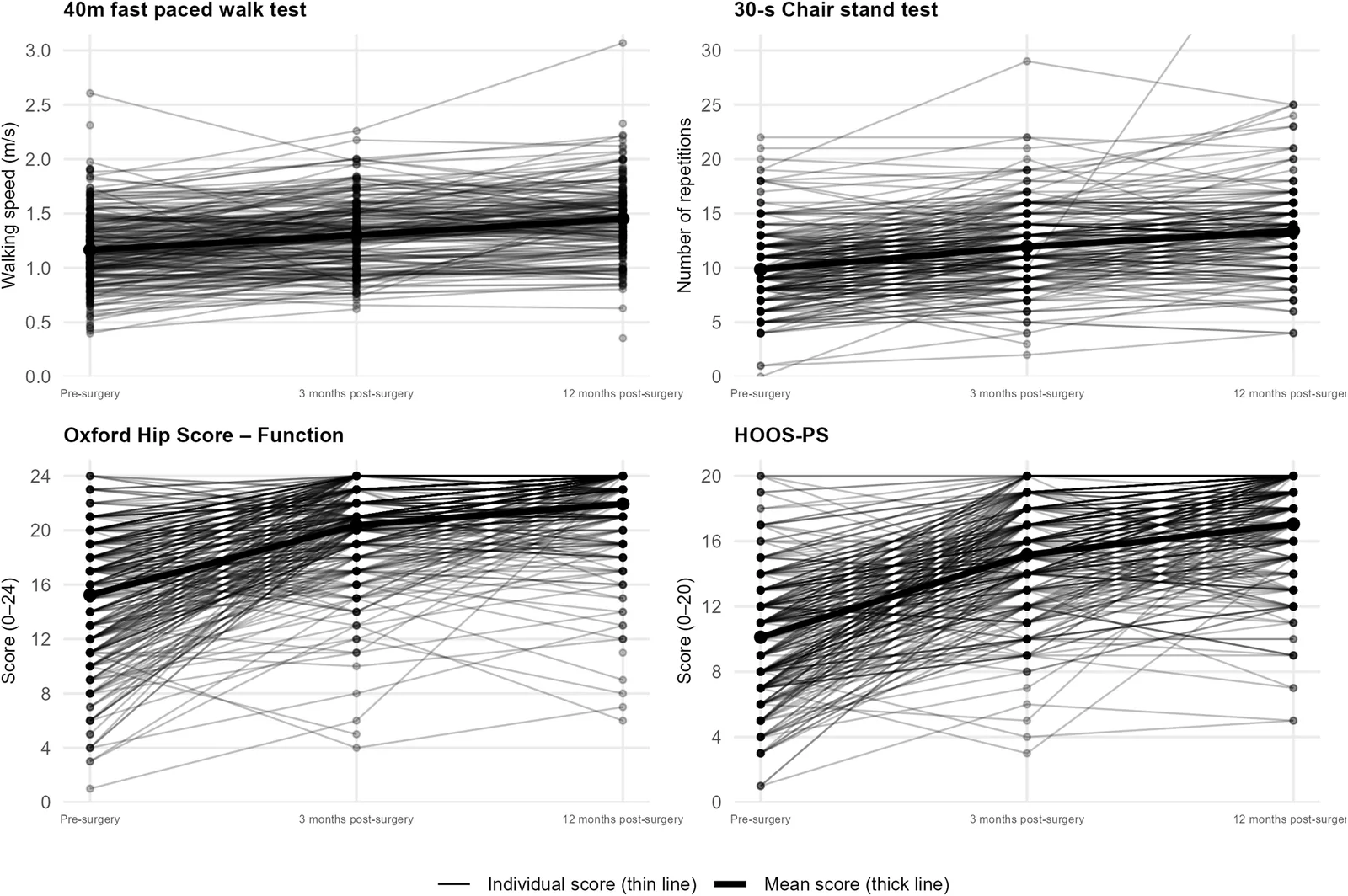
**Changes in outcomes of individuals undergoing a total hip arthroplasty taking part in the PaTIO-study** Figure legend. HOOS-PS: Hip Disability and Osteoarthritis Score – Physical function Shortform.

Fig. 3 shows the average change and individual trajectories for the primary outcomes in the TKA group. From pre-surgery to the 12-month follow-up, patients improved on average by 18% (0.18 m/s, SD 0.29) in gait speed on the 40 m FPWT, by 31% (2.3 repetitions, SD 3.5) on the 30-s chair-stand test, by 44% (4.1 points, SD 3.7) on the OKS function subscale, and by 50% (7.8 points, SD 6.2) on the KOOS-PS.

**Fig. 3:**
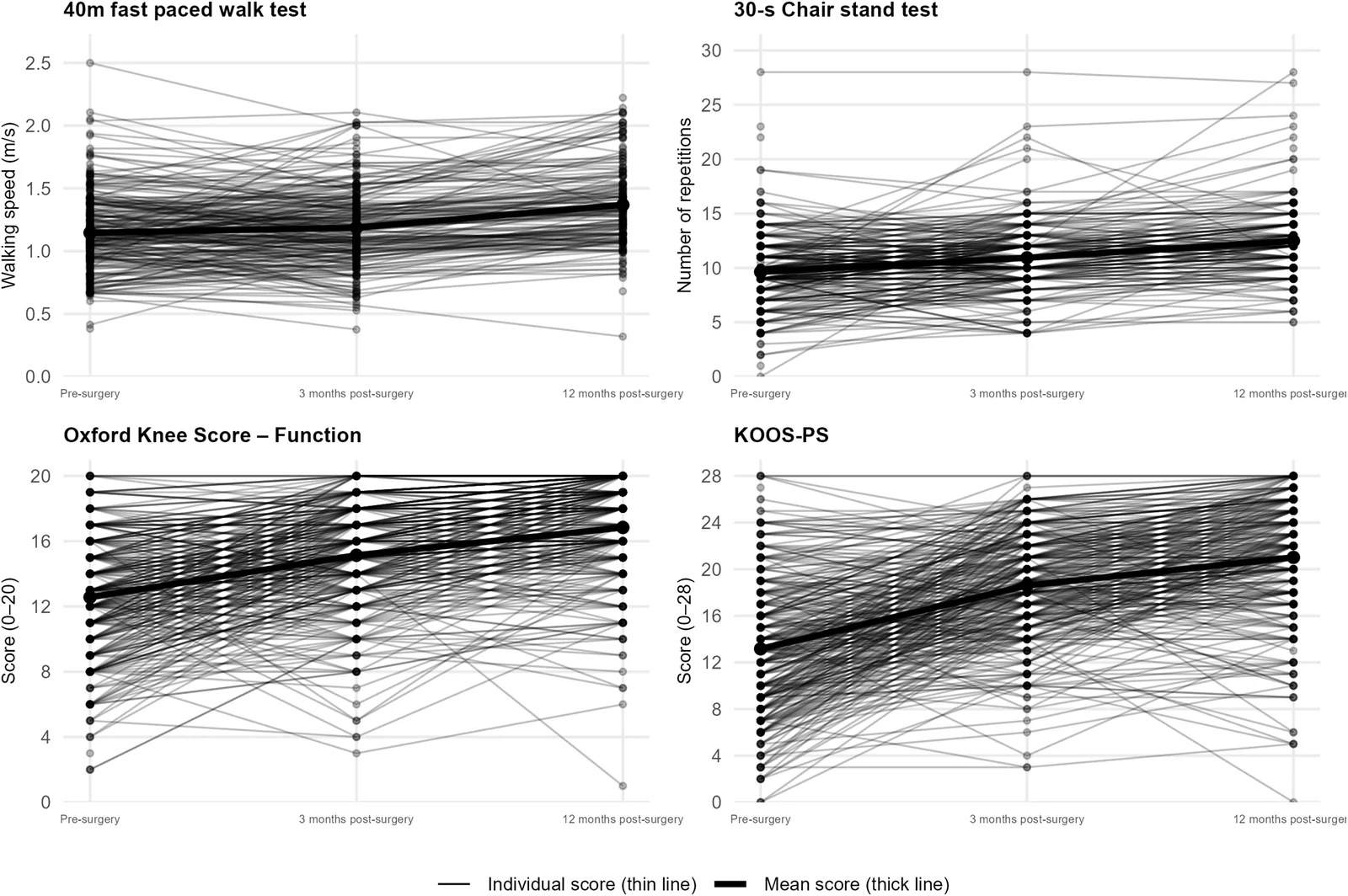
**Changes in outcomes of individuals undergoing a total knee arthroplasty taking part in the PaTIO-study** Figure legend. KOOS-PS: Knee Injury and Osteoarthritis Score – Physical function Shortform.

### 3.4 Random intercept crossed-lagged panel models

Path diagrams with standardized effects for each model are presented in Supplementary File 2. Fig. 4 provides an example of a path diagram showing standardized effects between the 40 m FPWT and the HOOS-PS in the THA group. Below, both standardized and unstandardized effects are described for the intervals between the preoperative and 3-month postoperative time points, and between the 3- and 12-month postoperative time points.

**Fig. 4:**
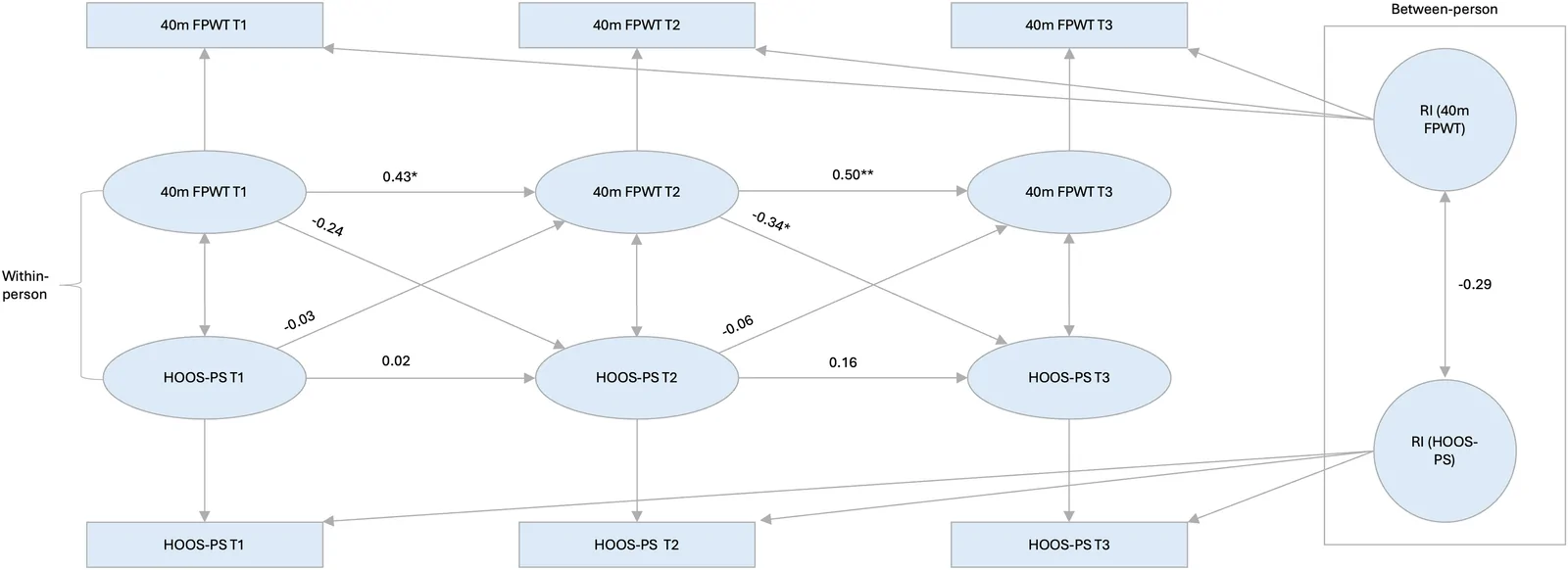
**Random intercept cross-lagged panel model of 40m FPWT and HOOS-PS** Figure legend of Random Intercept Cross lagged panel model showing standardized beta's of 40 m FPWT and HOOS-PS over 3 measurement occasions. Model is corrected for confounding of baseline variables on the observed variables at each assessment. Confounders included: age, sex, body mass index, living status (alone/together), preoperative pain scores, American society of Anaesthesiologists classification, and duration of complaints. 40 m FPWT = 40 m fast paced walk test, HOOS-PS = Hip disability and Osteoarthritis Outcome Score-Physical functioning Shortform, T1 = time 1 (pre-surgery), T2 = time 2 (3-months post-surgery, T3 = time 3 (12-months post-surgery), RI = Random Intercept, ∗ = p < 0.05, ∗∗ = p < 0.01.

#### 3.4.1 Effect of pre-surgery capacity on self-perceived performance at 3-months post-surgery

Overall, no cross-lagged effects were observed between the pre-surgery capacity and 3-month post-surgery self-perceived performance in either the THA or TKA groups, suggesting that pre-surgery levels of capacity do not influence short term post-surgery levels of self-perceived performance (Table 4).

**Table 4:** Crossed lagged effects between performance-based tests outcomes and self-perceived physical functioning.

	Total hip arthroplasty	Total knee arthroplasty
	Standardized coefficient (95% CI)	Standardized coefficient (95% CI)
**Effect of pre-surgery capacity on self-perceived performance at 3-months post-surgery**		
40 m fast-paced walk test **T1** → OHS/OKS **T2**	0.01 (−0.28, 0.31)	0.08 (−0.26, 0.42)
40 m fast-paced walk test **T1** → HOOS-PS/KOOS-PS **T2**	−0.24 (-0.54, 0.05)	−0.05 (−0.54, 0.43)
30-s Chair stand test **T1** → OHS/OKS **T2**	0.04 (−0.25, 0.34)	0.03 (−0.25, 0.31)
30-s Chair stand test **T1** → HOOS-PS/KOOS-PS **T2**	−0.07 (−0.36, 0.22)	0.11 (−0.28, 0.49)
**Effect of pre-surgery self-perceived performance on capacity at 3-months post-surgery**		
OHS/OKS **T1** → 40 m fast paced walk test **T2**	0.08 (−0.16, 0.33)	0.03 (−0.21, 0.28)
OHS/OKS **T1** → 30-s chair stand test **T2**	−0.03 (−0.22, 0.16)	−0.10 (−0.38, 0.18)
HOOS-PS/KOOS-PS **T1** → 40 m fast paced walk test **T2**	−0.03 (−0.25, 0.18)	−0.03 (−0.26, 0.20)
HOOS-PS/KOOS-PS **T1** → 30-s chair stand test **T2**	0.04 (−0.15, 0.24)	0.15 (−0.10, 0.41)
**Effect of capacity at 3 months post-surgery on self-perceived performance at 12 months post-surgery**		
40 m fast-paced walk test **T2** → OHS/OKS **T3**	0.19 (−0.11, 0.49)	**0.24 (0.05, 0.43)**
40 m fast-paced walk test **T2** → HOOS-PS/KOOS-PS **T3**	**0.34 (0.05, 0.63)**	**0.29 (0.11, 0.47)**
30-s Chair stand test **T2** → OHS/OKS **T3**	0.14 (−0.13, 0.6)	0.17 (−0.02, 0.36)
30-s Chair stand test **T2** → HOOS-PS/KOOS-PS **T3**	−0.15 (−0.37, 0.08)	−0.13 (−0.35, 0.09)
**Effect of self-perceived performance at 3 months post-surgery on capacity at 12 months post-surgery**		
OHS/OKS **T2** → 40 m fast paced walk test **T3**	−0.11 (−0.31, 0.10)	−0.01 (−0.17, 0.14)
OHS/OKS **T2** → 30-s chair stand test **T3**	0.07 (−0.15, 0.28)	−0.08 (−0.26, 0.10)
HOOS-PS/KOOS-PS **T2** → 40 m fast paced walk test **T3**	−0.06 (−0.26, 0.14)	−0.02 (−0.17, 0.13)
HOOS-PS/KOOS-PS **T2** → 30-s chair stand test **T3**	−0.04 (−0.23, 0.16)	**−0.17 (−0.31, −0.00)**

Table legend. RI = random intercept, OHS/OKS = Oxford Hip/Knee Score function subscale, HOOS-PS = Hip disability Osteoarthritis Outcome Score-Physical functioning Shortform, KOOS-PS = Knee injury Osteoarthritis Outcome Score-Physical functioning Shortform, T2 = time 2 (3 months post total hip/knee arthroplasty), T3 time 3 = (12 months post total hip/knee arthroplasty).

#### 3.4.2 Effect of pre-surgery self-perceived performance on capacity at 3-months post-surgery

Similarly, no cross-lagged effects were observed between the pre-surgery self-perceived performance and 3-month post-surgery capacity in either the THA or TKA groups, suggesting that pre-surgery levels of self-perceived performance do not influence short term post-surgery levels of physical capacity (Table 4).

#### 3.4.3 Effect of capacity at 3 months post-surgery on self-perceived performance at 12 months post-surgery

In the THA group, the 40 m FPWT at 3 months post-surgery had no effect on OHS scores at 12 months but did have an effect (β 0.34 95% CI 0.05, 0.63**)** on HOOS-PS scores. For each m/s an individual walked above their personal average gait speed at 3 months post-surgery, on average, they scored 3.8 points (95% CI: 0.6, 6.9) above their personal average HOOS-PS score at 12 months post-surgery. This suggests that patients walking faster than their personal average at 3 months report better than their personal average self-perceived performance (only HOOS-PS) at 12 months.

In the TKA group, the 40 m FPWT showed effects on both the OKS (β 0.24 95% CI 0.05, 0.43**)** and KOOS-PS (β 0.29 95% CI 0.11, 0.47) scores. For each m/s individuals walked above their personal average gait speed at 3 months post-surgery, on average, they scored 2.6 points (95% CI 0.5, 4.6) above their personal average OKS function score and 5.0 points (95% CI 1.8, 8.2) above their personal average KOOS-PS score. Again, suggesting that individuals with a TKA who walked faster than their personal average at 3 months report better than their personal average self-perceived performance (only KOOS-PS) at 12 months.

In both the THA and TKA group, the 3-month post-surgery 30-s CST showed no significant crossed lagged effects with both the OKS and KOOS-PS at 12 months post-surgery. Indicating that early postoperative levels of lower extremity strength do not influence self-perceived performance at 12 months within individuals following THA and TKA.

#### 3.4.4 Effect of self-perceived performance at 3 months post-surgery on capacity at 12 months post-surgery

Only the KOOS-PS score at 3 months post-surgery showed a negative cross lagged effect (β −0.17 95% CI -0.31, −0.00,**)** on the 12-month 30-s CST-score. Each point an individual scored above their personal average KOOS-PS score at 3 months, their sit-to-stand performance decreased by 0.1 repetitions (95% CI 0.0, 0.3) below their personal average 30s CST performance at 12 months. Indicating that self-perceived performance above an individual's personal average at 3 months is followed by a negligibly lower than personal average lower extremity strength at 12 months.

## 4 Discussion

The present study showed no effects between capacity (40 m FPWT and 30-s CST) and self-perceived performance (OHS/OKS and HOOS-PS/KOOS-PS) from pre-surgery to 3-month post-surgery within individuals. Preoperative capacity had no effect on self-perceived performance at 3 months post-surgery, and preoperative self-perceived performance had no effect on capacity at 3 months post-surgery in both THA and TKA. From 3-months to twelve months post-surgery individuals who walked faster than their personal average gait speed at 3-month post-surgery reported better than their personal average self-perceived performance at 12-months in both THA (only HOOS-PS) and TKA. No other meaningful longitudinal effects were observed.

### 4.1 Comparison to earlier findings

These findings are consistent with earlier studies reporting that capacity and self-perceived performance develop independently over time. In THA, Dayton et al. observed that early postoperative declines in Timed Up & Go, stair-climb, and 6-min walk performance occurred despite improvements in HOOS function scores, with no significant correlations between pre-to postoperative changes [16]. Similarly, Naili et al. found that HOOS outcomes one year after THA did not differ between patients classified as improved versus unchanged based on performance thresholds [17]. In TKA, Stevens-Lapsley et al. reported no consistent correlations between changes in 6-min walk distance and KOOS-Activities of Daily Living scores [18], while Mizner et al. demonstrated that capacity worsened acutely after surgery without parallel declines in self-perceived performance [19]. Comparable dissociations have also been described in mixed THA/TKA cohorts during early recovery [20,21]. By using a within-person longitudinal model, our findings support this pattern even at the individual level. However, patients who walked faster than their personal average gait speed at 3 months report better than their personal average perceived performance at 12 months.

The longitudinal effect from gait speed to subsequent self-perceived performance may be explained by the construct overlap between the 40 m FWPT and the PROM items. The 40 m FPWT is timed over 4 × 10 m and requires repeated turns around cones/markers, capturing not only fast walking but also change-of-direction. This aligns closely with the HOOS-PS item *twisting/pivoting on the loaded leg* and with the KOOS-PS item *twisting/pivoting on the injured knee* which may explain the stronger effects observed in these measures. Such dynamic mobility items are not explicitly mentioned in the Oxford function components we analysed. Furthermore, gait performance is a strong global predictor of self-perceived functioning in community dwelling adults [45]. It has even been shown to be associated to self-reported difficulty in rising from a chair [46], an item included in both the OHS/OKS and HOOS-PS/KOOS-PS. The lack of an effect of gait speed on the OHS may partly be explained by a ceiling effect, as most patients showed substantial improvement within the first three months after surgery, after which little additional improvement was observed at the individual level.

The absence of an effect between chair stand performance and subsequent self-perceived performance may be explained by the influence of additional factors on the related items. Although the OHS/OKS and HOOS-PS/KOOS-PS include several items that relate to chair raise performance, such as *squatting, getting in/out of a car* and *getting out of bed*. These items depend not only on lower extremity strength but are also affected by joint range of motion [46]. Moreover, perceived difficulty in rising from a chair is associated to health-related characteristics that reflect frailty even after accounting for chair rise performance [46], indicating that perceived limitation is influenced by more than task capacity alone. This may also explain the somewhat counterintuitive but clinically negligible effect of self-perceived performance at 3 months on chair rise performance at 12-months.

### 4.2 Strengths and limitations

Strengths of this study include the relatively large sample size and the use of a random-intercept cross-lagged panel model, which allowed us to examine the temporal ordering of capacity and self-perceived performance and extend beyond cross-sectional associations or concurrent changes. However, some limitations should be considered when interpreting our results.

First, although the random intercepts account for stable between-person differences including stable psychosocial factors, time varying psychosocial factors were not explicitly modelled. Factors such as changes in depression, self-efficacy or psychosocial distress may influence both capacity and self-perceived performance over time [47,48] and could therefore have contributed to the observed within-person effects [49].

Second, our measurements of capacity were limited to gait speed and lower-extremity strength. While these are important domains, they do not fully capture capacity after THA/TKA; recommended performance-based assessment batteries also include tests reflecting transfer capability, balance/mobility, and endurance (e.g., timed up-and-go, stair climbing, 6-min walk) [11], which may show different longitudinal relations with PROMs. Consequently, our findings should not be generalized to capacity domains that were not measured.

Third, PROM measurement characteristics may have influenced our results. The strongest longitudinal effects were observed for HOOS-PS/KOOS-PS, yet a systematic review reported inconsistent evidence for their content validity and raised cautioned against their use for evaluating change [47]. Thus, the observed effects could partly reflect instrument-specific measurement limitations, such as reduced variability, rather than actual changes in physical functioning. However, we also included the OHS/OKS, which have shown good measurement properties in both THA and TKA [48]. Therefore, when effects between capacity and self-perceived performance are observed in one PROM but not the other, these discrepancies may reflect instrument-specific measurement characteristics rather than true differences in the underlying relationship between capacity and self-perceived performance. Additionally, response shift may have further attenuated effects. Response shift occurs when the meaning of one's self-evaluation changes due to recalibration, reprioritization or reconceptualization of the construct being measured [49]. PROM-scores may remain unchanged because the way patients interpreted or valued functional limitations shifted over time, whereas performance-based capacity measures are unaffected by such cognitive adaptations.

Finally, our results may have been influenced by the COVID-19 pandemic, during which original trial data was collected. The pandemic has been associated with increased levels of psychological distress, anxiety, depressive symptoms and reduced mental well-being [50]. Psychological wellbeing has shown to influence self-perceived performance, independent of capacity following orthopaedic surgery [51]. However, previous research has shown no clinically relevant differences in self-perceived performance between patients who had received a THA or TKA before or during the pandemic [52]. Therefore, although pandemic-related psychological changes cannot be entirely excluded, it is unlikely that they substantially influenced are findings.

## 5 Conclusion

From pre-surgery to 3 months post-surgery capacity and self-perceived performance showed no effects within individuals following THA and TKA. From 3 to 12 months post-surgery faster than personal average gait speed was followed by higher than personal average self-perceived performance, whereas lower extremity strength did not influence subsequent self-perceived performance. Self-perceived performance did not influence either subsequent gait speed or lower extremity strength. Clinically, this implies that gait speed may represent a relevant rehabilitation target in the early postoperative phase to improve long term self-perceived performance. However, clinicians should be cautious in assuming that improvements in lower extremity strength will translate into better perceived performance. Future research should examine whether other domains of capacity are prospectively related to self-perceived performance and investigate the potential influence of time-varying psychosocial factors and response shift on these relationships.

## Contributions

**Dennis J. van den Berg**: conceptualization, data curation, formal analysis, funding acquisition, investigation, methodology, project administration, software, validation, visualization, writing – original draft.

**Esther T. Maas**: conceptualization, formal analysis, funding acquisition, methodology, validation, visualization, writing – original draft, supervision.

**Thea P.M. Vliet Vlieland**: conceptualization, funding acquisition, supervision, writing – review & editing.

**Henri Kiers**: conceptualization, writing – review & editing.

**Raymond W.J.G Ostelo**: conceptualization, funding acquisition, methodology, writing – review & editing.

**Max Reijman**: conceptualization, funding acquisition, writing – review & editing.

**Maaike G.J. Gademan**: conceptualization, data curation, formal Analysis, funding acquisition, investigation, methodology, validation, visualization, writing – original draft, supervision.

## Declaration of generative AI in scientific writing

During the preparation of this work, the author(s) used ChatGPT (OpenAI) and Le Chat (Mistral AI) to improve the readability and language of the manuscript. After using these tools, the authors reviewed and edited the content as needed and take full responsibility for the content of the publication.

## Role of funding source

This publication is part of the Doctoral Grant for Teachers research programme with file number 023.021.045, which is financed by the Dutch Research Council (NWO). The PaTIO study was financially supported by Leading the Change (80–85009–98-1002) and co-financed by the Dutch Orthopaedic Association. The funders had no role in study design, data collection and analysis, the decision to publish, or the preparation of this manuscript.

## Competing interests

The authors are unaware of any competing interests.
