# Supplementary material for: The longitudinal relationship between physical capacity and self-perceived physical functioning after total hip and knee arthroplasty
Source: Osteoarthr Cartil Open. 2026 Sep 23;8(4):100880. doi: 10.1016/j.ocarto.2026.100880 (PMC13653965; doi:10.1016/j.ocarto.2026.100880)
Supplement: Supplementary file 1 [file mmc1.docx]

**Supplementary file 1**

|  | Total Hip arthroplasty | | | Total knee arthroplasty | | |
| --- | --- | --- | --- | --- | --- | --- |
|  | All Cases (n=312) | Complete (n=158) | Incomplete (n=154) | All cases (n=312) | Complete (n=143) | Incomplete  (n=169) |
| Age, mean (SD) | 66.6 (8.9) | 66.4 (8.2) | 66.7 (9.5) | 66.9 (8.2) | 66.5 (7.8) | 67.3 (8.6) |
| Sex |  | | |  | | |
| - Male, no. (%) | 147 (47) | 72 (46) | 75 (48) | 140 (45) | 74 (52) | 66 (40) |
| - Female, no. (%) | 165 (53) | 86 (54) | 79 (52) | 169 (54.69) | 69 (48) | 100 (60) |
| BMI, mean (SD) | 27.1 (4.5) | 26.9 (4.1) | 27.2 (4.9) | 29.4 (4.9) | 29.2 (5) | 29.6 (4.9) |
| Living status, |  | | |  | | |
| - Living alone, no. (%) | 67(22) | 27 (17) | 40 (27) | 52 (17) | 23 (16) | 29 (18) |
| - Living together, no. (%) | 237 (78) | 128 (83) | 109 (73) | 253 (83) | 117 (84) | 136 (82) |
| Years of complaints, mean (SD) | 4.9 (5.8) | 4.8(6.1) | 5.1 (5.4) | 9.9 (9.1) | 10.8 (10) | 9.1 (8.3) |
| ASA |  | | |  | | |
| - I, no. (%) | 60 (19) | 34 (22) | 26 (17) | 53 (17) | 28 (20) | 25 (15) |
| - II, no. (%) | 192 (62) | 102 (65) | 89 (59) | 189 (61) | 82 (57) | 107 (64) |
| - III, no. (%) | 55 (18) | 21 (13) | 34 (23) | 68 (22) | 33 (23) | 35 (21) |
| Pain during activity, mean (SD) | 3.9 (3) | 3.9 (2.9) | 3.9 (3.1) | 6.7 (2) | 6.7 (2) | 6.7 (2) |
| OHS/OKS function subscale raw score |  | | |  | | |
| - Preoperative,   mean (SD) | 15.3 (8.3) | 16 (4.6) | 14.5 (4.8) | 12.6 (3.8) | 13.2 (3.3) | 12.1 (4.1) |
| - 3 Months post-surgery, mean (SD) | 20.4 (3.5) | 20.7 (3) | 20.1 (4) | 15.1 (3.4) | 15.3 (3.2) | 15 (3.5) |
| - 12 months post-surgery, mean (SD) | 21.9 (3.2) | 22.3 (2.6) | 21.5 (3.8) | 16.8 (3.1) | 17.1 (3) | 16.6 (3.2) |
| HOOS-PS/KOOS-PS score |  | | |  | | |
| - Preoperative,   mean (SD) | 10.1 (3.8) | 10.5 (3.9) | 9.7 (3.7) | 13.2 (5.9) | 13.3 (5.6) | 13 (6.1) |
| - 3 Months post-surgery, mean (SD) | 15.2 (3.3) | 15.5 (3) | 14.8 (3.6) | 18.6 (4.8) | 18.4 (4.7) | 18.8 (4.9) |
| - 12 months post-surgery, mean (SD) | 17 (3.1) | 17.3 (2.9) | 16.8 (3.4) | 21 (5.3) | 21.2 (5.3) | 20.8 (5.3) |

**Sample demographics and outcomes grouped by completeness of performance-based tests across all occasions**
*Table legend. n = number, BMI = body mass index, SD = standard deviation, ASA = American Society of Anaesthesiologists, HOOS-PS/KOOS-PS = Hip Disability/Knee Injury Osteoarthritis Outcome Score, OHS/OKS = Oxford Hip/Knee Score, 40m FPWT = 40-metre Fast Paced Walking Test, 30-s CST = 30-seconds Chair Stand Test.*
