# Supplementary material for: The longitudinal relationship between physical capacity and self-perceived physical functioning after total hip and knee arthroplasty
Source: Osteoarthr Cartil Open. 2026 Sep 23;8(4):100880. doi: 10.1016/j.ocarto.2026.100880 (PMC13653965; doi:10.1016/j.ocarto.2026.100880)
Supplement: Supplementary file 2 [file mmc2.docx]

**Supplementary file 2**

**Figure S1. Random intercept crossed-lagged panel model of 40m FPWT and OHS**
Figure legend of Random Intercept Cross lagged panel model showing standardized beta’s of 40m FPWT and OKS over 3 measurement occasions. Model is corrected for confounding of baseline variables on the observed variables at each assessment. Confounders included: age, sex, body mass index, living status (alone/together), preoperative pain scores, American society of Anaesthesiologists classification, and duration of complaints. 40m FPWT = 40 metre fast paced walk test, OKS = Oxford Knee Score function subscale, T1 = time 1 (pre-surgery), T2 = time 2 (3-months post-surgery, T3 = time 3 (12-months post-surgery), RI = Random Intercept, * = p < 0.05, ** = p < 0.01

**Figure S2. Random intercept cross-lagged panel model of 40m FPWT and HOOS-PS**
Figure legend of Random Intercept Cross lagged panel model showing standardized beta’s of 40m FPWT and HOOS-PS over 3 measurement occasions. Model is corrected for confounding of baseline variables on the observed variables at each assessment. Confounders included: age, sex, body mass index, living status (alone/together), preoperative pain scores, American society of Anaesthesiologists classification, and duration of complaints. 40m FPWT = 40 metre fast paced walk test, HOOS-PS = Hip disability and Osteoarthritis Outcome Score-Physical functioning Shortform, T1 = time 1 (pre-surgery), T2 = time 2 (3-months post-surgery, T3 = time 3 (12-months post-surgery), RI = Random Intercept, * = p < 0.05, ** = p < 0.01.

**Figure S3. Random intercept cross-lagged panel model of 30-s CST and OHS**

Figure legend of Random Intercept Cross lagged panel model showing standardized beta’s of 30-s CST and OHS over 3 measurement occasions. Model is corrected for confounding of baseline variables on the observed variables at each assessment. Confounders included: age, sex, body mass index, living status (alone/together), preoperative pain scores, American society of Anaesthesiologists classification, and duration of complaints. 30-s CST= 30 seconds Chair Stand Test, OKS= Oxford Hip Score function subscale, T1 = time 1 (pre-surgery), T2 = time 2 (3-months post-surgery, T3 = time 3 (12-months post-surgery), RI = Random Intercept, * = p < 0.05, ** = p < 0.01.

**Figure S4. Random intercept cross-lagged panel model of the 30-s CST and HOOS-PS**

Figure legend of Random Intercept Cross lagged panel model showing standardized beta’s of 30-s CST and HOOS-PS over 3 measurement occasions. Model is corrected for confounding of baseline variables on the observed variables at each assessment. Confounders included: age, sex, body mass index, living status (alone/together), pre-operative pain scores, American society of Anaesthesiologists classification, and duration of complaints. 30-s CST= 30 seconds Chair Stand Test, HOOS-PS = Hip disability and Osteoarthritis Outcome Score-Physical functioning Shortform, T1 = time 1 (pre-surgery), T2 = time 2 (3-months post-surgery, T3 = time 3 (12-months post-surgery), RI = Random Intercept, * = p < 0.05, ** = p < 0.01.

**Figure S5. Random intercept crossed-lagged panel model of 40m FPWT and OKS**
Figure legend of Random Intercept Cross lagged panel model showing standardized beta’s of 40m FPWT and OKS over 3 measurement occasions. Model is corrected for confounding of baseline variables on the observed variables at each assessment. Confounders included: age, sex, body mass index, living status (alone/together), preoperative pain scores, American society of Anaesthesiologists classification, and duration of complaints. 40m FPWT = 40 metre fast paced walk test, OKS = Oxford Knee Score function subscale, T1 = time 1 (pre-surgery), T2 = time 2 (3-months post-surgery, T3 = time 3 (12-months post-surgery), RI = Random Intercept, * = p < 0.05, ** = p < 0.01

**Figure S6. Random intercept cross-lagged panel model of 40m FPWT and KOOS-PS**

Figure legend of Random Intercept Cross lagged panel model showing standardized beta’s of 40m FPWT and KOOS-PS over 3 measurement occasions. Model is corrected for confounding of baseline variables on the observed variables at each assessment. Confounders included: age, sex, body mass index, living status (alone/together), preoperative pain scores, American society of Anaesthesiologists classification, and duration of complaints. 40m FPWT = 40 metre fast paced walk test, KOOS-PS = Knee injury and Osteoarthritis Outcome Score-Physical functioning Shortform, T1 = time 1 (pre-surgery), T2 = time 2 (3-months post-surgery, T3 = time 3 (12-months post-surgery), RI = Random Intercept, * = p < 0.05, ** = p < 0.01.

**Figure S7. Random intercept cross-lagged panel model of 30-s CST and OKS**

Figure legend of Random Intercept Cross lagged panel model showing standardized beta’s of 30-s CST and OKS over 3 measurement occasions. Model is corrected for confounding of baseline variables on the observed variables at each assessment. Confounders included: age, sex, body mass index, living status (alone/together), preoperative pain scores, American society of Anaesthesiologists classification, and duration of complaints. 30-s CST= 30 seconds Chair Stand Test, OKS= Oxford Knee Score function subscale, T1 = time 1 (pre-surgery), T2 = time 2 (3-months post-surgery, T3 = time 3 (12-months post-surgery), RI = Random Intercept, * = p < 0.05, ** = p < 0.01.

**Figure S8. Random intercept cross lagged-panel model of the 30-s CST and KOOS-PS**

Figure legend of Random Intercept Cross lagged panel model showing standardized beta’s of 30-s CST and KOOS-PS over 3 measurement occasions. Model is corrected for confounding of baseline variables on the observed variables at each assessment. Confounders included: age, sex, body mass index, living status (alone/together), preoperative pain scores, American society of Anaesthesiologists classification, and duration of complaints. 30-s CST= 30 seconds Chair Stand Test, KOOS-PS = Knee injury and Osteoarthritis Outcome Score-Physical functioning Shortform, T1 = time 1 (pre-surgery), T2 = time 2 (3-months post-surgery, T3 = time 3 (12-months post-surgery), RI = Random Intercept, * = p < 0.05, ** = p < 0.01.
